# Understanding and predicting disease relationships through similarity fusion

**DOI:** 10.1093/bioinformatics/bty754

**Published:** 2018-08-30

**Authors:** Erin Oerton, Ian Roberts, Patrick S H Lewis, Tim Guilliams, Andreas Bender

**Affiliations:** 1 Centre for Molecular Informatics, Department of Chemistry, University of Cambridge, Cambridge, UK; 2 Healx Ltd, Park House, Castle Park, Cambridge, UK

## Abstract

**Motivation:**

Combining disease relationships across multiple biological levels could aid our understanding of common processes taking place in disease, potentially indicating opportunities for drug sharing. Here, we propose a similarity fusion approach which accounts for differences in information content between different data types, allowing combination of each data type in a balanced manner.

**Results:**

We apply this method to six different types of biological data (ontological, phenotypic, literature co-occurrence, genetic association, gene expression and drug indication data) for 84 diseases to create a ‘disease map’: a network of diseases connected at one or more biological levels. As well as reconstructing known disease relationships, 15% of links in the disease map are novel links spanning traditional ontological classes, such as between psoriasis and inflammatory bowel disease. 62% of links in the disease map represent drug-sharing relationships, illustrating the relevance of the similarity fusion approach to the identification of potential therapeutic relationships.

**Availability and implementation:**

Freely available under the MIT license at https://github.com/e-oerton/disease-similarity-fusion

**Supplementary information:**

Supplementary data are available at *Bioinformatics* online.

## 1 Introduction

Establishing relationships between diseases increases our understanding of disease biology, aiding the identification of shared mechanisms or development of new treatments, for example through drug repurposing. The identification of novel relationships between diseases is therefore of great biological and pharmacological interest. Traditionally, diseases have been grouped based on their symptoms, the part of the body that they affect, or their etiology (Wang *et al.*, 2017), but the development of novel bioinformatics technologies has allowed diseases to be quantified and related in many new ways. In particular, (-omics) data allows greater understanding of what is taking place in disease at a molecular level, enabling diseases to be related through disease-associated genes (Cheng *et al.*, 2014; Goh *et al.*, 2007; Sun *et al.*, 2014b), protein interaction networks (Menche *et al.*, 2015), gene expression (Hu and Agarwal, 2009; Suthram *et al.*, 2010; Yang *et al.*, 2015), pathways (Li and Agarwal, 2009) and biological processes (Mathur and Dinakarpandian, 2012). Rather than examining each of these different data types in isolation, recent studies have related diseases by considering multiple data types simultaneously. These data integration approaches can provide a more comprehensive understanding of disease, potentially reflecting interactions between the different layers of the biological system (Ritchie *et al.*, 2015) where links at one layer (e.g. genetic variance) are associated with changes at another layer (e.g. gene expression or phenotype). Recent examples have demonstrated how this can be achieved through the use of heterogeneous networks, such as the DiseaseConnect web server developed by Liu *et al.* (2014), or through matrix factorization approaches, such as that presented by Žitnik *et al.* (2013).

However, these approaches do not quantify the overall strength of the relationship across multiple levels. Defining a measure of disease similarity that takes into account multiple data types is not straightforward, as such a measure must consider differences between properties such as information content (Gligorijević and Pržulj, 2015). Sun *et al.* (2014a, b) evaluated disease similarity by defining a feature vector for each disease in which every element (genes, chemicals, pathways and GO terms) was weighted according to its information content. The downside of this approach is that it requires an entry for each entity in the feature universe, needing a feature vector of tens of thousands of dimensions to represent just four spaces. Computing similarity across multiple spaces by this approach therefore does not scale readily to large numbers of feature spaces.

In this work, we address this issue by translating the feature vectors in each space into pairwise disease similarities, thus capturing disease relationships in a lower-dimensional space before performing the integration step to define an overall measure of similarity. This ‘similarity fusion’ approach has been successfully applied to integrate data in drug repositioning (Gottlieb *et al.*, 2011; Wang *et al.*, 2013), gene prioritization (Chen *et al.*, 2011) and patient subtyping and survival analysis (Speicher and Pfeifer, 2015; Wang *et al.*, 2014). Yet there have been few applications of this approach to quantify disease similarity. In one study, disease similarities were computed by integrating literature-based similarity of diseases with topology-based similarity of their associated genes (Li *et al.*, 2016); more recent work related diseases through ‘meta-correlation’, combining similarity amongst gene expression and electronic health record profiles of diseases (Haynes *et al.*, 2017). Another study integrated similarity in nine different spaces according to a pre-defined ‘importance’, with the resulting relationships weighted towards genetic similarities (Jalili *et al.*, 2016). Although the relative ‘importance’ of each relationship type naturally depends on the context in which the map is used, no study has yet defined a general method for the combination of multiple disease similarities in an unbiased manner. In particular, unbiased combination of spaces requires consideration of the underlying distributions of similarity in each space. To address this issue, we propose the use of quantile normalization to adjust the similarity distributions, enabling balanced comparison and combination of disease similarities across multiple spaces.

In summary, we define a single measure of disease similarity over six different data types—ontological, phenotypic, literature co-occurrence, genetic association, gene expression and drug data—and propose a framework for fusion of these similarities using quantile normalization. The fused similarities define a disease map: a network of diseases connected at one or more biological levels. We explore the unexpected disease links revealed by this approach and their relation to existing disease classifications and drug-sharing relationships. 

## 2 Materials and methods

### 2.1 Disease dataset construction

**Fig. 1.**
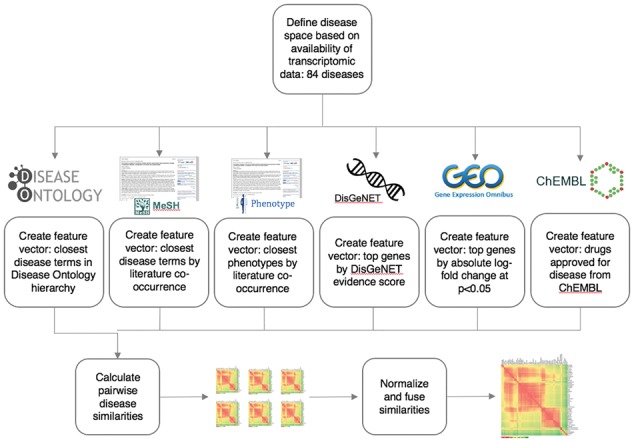
Disease similarity fusion workflow. Disease data from six different ‘feature spaces’ are transformed into symmetric similarity matrix representations. Feature sets for each disease are formed of the approximately 100 top features in each space, although the exact number varies depending on the available data. Similarity matrices representing each individual feature space are then normalized and combined into a single fused similarity matrix. The disease relationships represented by this matrix can be analyzed to find novel links between diseases or links which may represent drug-sharing opportunities

The disease dataset which formed the basis of this work was manually compiled by searching Gene Expression Omnibus (Barrett *et al.*, 2013) for common diseases and selecting those where patient-derived transcriptomic data were available. This resulted in a dataset of 84 diseases, some of which were closely related (e.g. asthma and allergic asthma; see Supplementary File S1). These diseases were mapped to the most closely matching disease terms in each space (e.g. ‘teratozoospermia’ may map to ‘azoospermia’ or simply ‘male infertility’, depending on what representation is available in each space; see Supplementary File S2 for details). Feature sets for each disease were then constructed in each of ontological, phenotypic, literature co-occurrence, genetic, transcriptomic and drug spaces using R version 3.3.2 (R Core Team, 2015). Figure 1 shows an overview of this process (see Supplementary Text S1 for details). The feature set size of phenotypic space was restricted to ∼21 by the dataset used; the feature set size of drug space was restricted by the number of drugs prescribed for each disease. For the remaining spaces, a feature set size of 100 was chosen, as this captured sufficient information in each space whilst not being overly large compared to the fixed-size feature spaces. The exact number may be slightly more or less than 100 for some diseases due to e.g. ties in the data (see Supplementary Text S2, Supplementary Table S1 and Supplementary Figs S1 and S2 for details and exploration of different feature set sizes).

### 2.2 Similarity fusion

Pairwise similarity scores between each of the 84 diseases were calculated based on the Jaccard index of their feature sets. In the case of transcriptomic data, where the sizes of the up- and down-regulated sets are unequal, the Jaccard score was calculated as a weighted mean of Jaccard scores for the two sets. As the distributions of similarity scores within each space are uneven, fusion of the raw similarity scores would cause those spaces with higher average scores to dominate the fused similarity. Even if the scores are normalized to the same sum, the fused similarities would still be affected by the differences in distribution of similarity values in each space (e.g. causing sparse spaces to dominate the fused scores at high similarities). We therefore applied quantile normalization (Bolstad *et al.*, 2003) to adjust the distributions of similarity scores towards each other, enabling comparison and combination of each space. Similarity values were quantile-normalized using the normalizeQuantiles function from limma (version 3.30.13), including adjustment for tied values. A single ‘fused’ similarity score was then computed by taking the mean of the individual quantile-normalized similarity values for each space, resulting in a 3486-dimensional similarity vector (or an 84 × 84 symmetric similarity matrix) forming the basis of the disease map. The map presented here is based on an unweighted mean of spaces, although the method allows the specification of weights in order to adjust the influence of each space on the fused similarities. An example of the similarity calculation for a pair of diseases is given in Supplementary Text S3.

### 2.3 Defining a significance threshold for disease similarity

To construct the disease map, we defined a threshold of significant similarity above which diseases are linked, based on 1000 random similarity matrices. Randomized feature vectors were constructed for each disease by sampling from the feature universe, defined as the union of all features in that space across all diseases in the dataset, according to their distribution (frequency) in the dataset. Random fused matrices were created from these random feature vectors as described in section 2.2. A comparison of the distribution of the random against the real fused similarity scores is given in Supplementary Figure S4. The 99.99th percentile of the random similarity scores (equivalently, the maximum similarity observed in 83% of the random matrices) was taken as the threshold of similarity above which diseases were considered to be linked. 6.9% of similarity values in the network were above this threshold. Cytoscape (Shannon *et al.*, 2003) was used for network visualization.

### 2.4 Evaluating the fused similarity scores

An initial evaluation of the fused similarity scores was carried out against an independent disease comorbidity dataset (Hidalgo *et al.*, 2009), which covers 938 of our 3486 disease pairs with 7 duplicate mappings; see Supplementary Text S1 for details.

Any disease-related evaluation data covering all diseases could also be used as a feature space, and so for detailed evaluation we used a ‘hold-out’ style measuring how well one feature space is represented in the remaining five. Firstly, drug approval information (obtained from ChEMBL as described above, although here we also included drugs in Phase III clinical trials) measures whether similarity between two diseases might indicate drug-sharing potential. Secondly, membership of Disease Ontology top-level classes measures how closely disease associations match established notions of clinical similarity. This was evaluated by training random forest classifiers on the pairwise similarity values, using the R package *randomForest* (Liaw and Wiener, 2002) with default parameters. To ensure availability of sufficient training data, DO class prediction was split into two binary tasks—membership of *disease of anatomical entity*, and membership of *disease of cellular proliferation*. Model performance was evaluated using stratified Monte Carlo cross-validation, with an 80–20 split into training and test sets. The true positive rate (TPR), false positive rate (FPR) and area under the ROC curve (AUROC) were calculated using the function *performance* from the package *ROCR* (Sing *et al.*, 2005) averaged over 1000 runs. In order to display ROC curves, TPR and FPR were averaged only over those runs where the mode average number of data points were recorded.

## 3 Results

### 3.1 Exploratory disease map analysis identifies existing and novel disease relationships

**Fig. 2.**
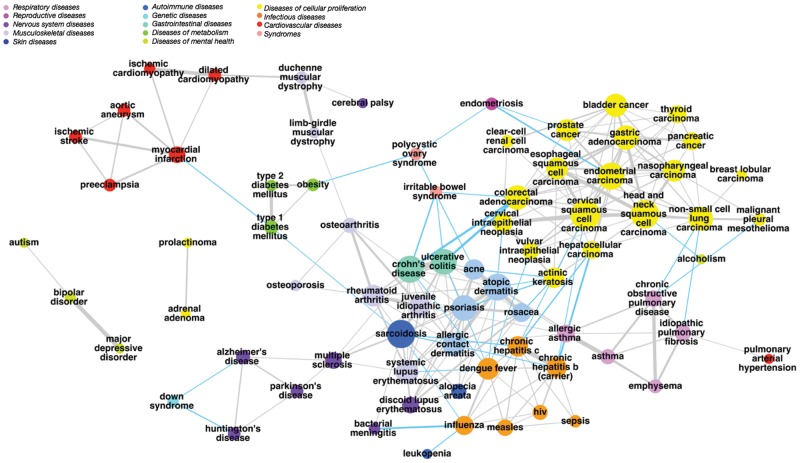
Disease map resulting from fused similarity scores. Connecting the most similar diseases defines a disease network, where edges represent similarity in multiple feature spaces. The network shown here is constructed from all six feature spaces and connects diseases not only within DO classes (the class ‘Disease of anatomical entity’ has been split into subclasses for clarity) but shows novel links (highlighted in blue) which are in different DO classes. More detail on these novel links is given in Supplementary Table S3. The network shown is based on a force-directed layout, with minor adjustments to node position for readability

The core of the disease mapping method is the conversion of feature spaces from high-dimensional feature sets into similarity vectors (Fig. 1), allowing comparison and combination of heterogeneous data types to create a ‘disease map’: a network of diseases that are linked at multiple biological levels. Links in the disease map represent similarities above a threshold of significance (calculated as described in Section 2.3) between the 84 diseases analyzed here, as shown in Figure 2. 81 of the 84 diseases are included in the map, with cystic fibrosis, teratozoospermia and placental malaria not showing any significant links to other diseases. Many links in the map correspond to the traditional ontological classes represented by the Disease Ontology (DO)—particularly within the DO classes *disease of cellular proliferation, disease by infectious agent* and *respiratory system disease*—but we additionally observed many novel links that span traditional disease categories, here defined as disease pairs which are not in the same top-level DO class. These novel links make up 15% of the links in the disease map. Many of the novel links represent diseases of distinct etiology which share similar features, such as actinic keratosis and psoriasis, or chronic obstructive pulmonary disease and malignant pleural mesothelioma (Supplementary Table S3).

If diseases linked in the map are pathologically related, they may be more likely to co-occur in the same patient. We therefore compared links in the disease map to disease comorbidities based on the medical records of 13 million patients (Hidalgo *et al.*, 2009). The 63 links for which comorbidity scores are available had a median relative risk (RR) of 2.35 (i.e. diseases are 2.35 times more likely to co-occur than expected by chance), compared to a median of 1.06 for the 875 scored pairs not linked in the disease map. 71% of these links co-occur in patients at a RR threshold above 1.5, compared to 27% of the non-linked pairs, or 2.6 times more often. At higher RR thresholds of 2 and 5, this ratio increases to 4.6 and 10.6, respectively. This relationship suggests that links in the disease map represent clinically relevant associations.

### 3.2 Case study: psoriasis

**Fig. 3.**
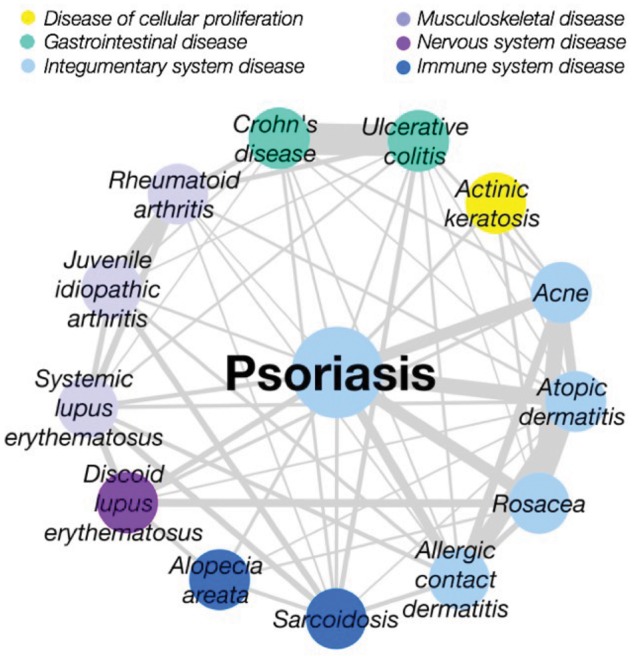
Diseases related to psoriasis. As well as known links to other skin diseases, psoriasis has links to a number of phenotypically distinct diseases with an autoimmune component, such as alopecia, arthritis and lupus, as well as inflammatory bowel diseases with which it shares genetic features related to drugs that can be used to treat both conditions. There is a high degree of interconnection amongst this group of diseases, which form one of the most densely connected areas in the network

The disease map also allows us to focus on connections of a disease of interest. As a case study, we examine psoriasis and its related diseases, which form a densely connected region of the map. Psoriasis is classified as a skin condition in Disease Ontology, but is known to have immune and hereditary components (Boehncke and Schön, 2015). This is reflected in the disease map, which links psoriasis to a number of autoimmune diseases as well as to other skin diseases (Fig. 3), such as the relationship between psoriasis and the inflammatory bowel diseases Crohn’s disease (CD) and ulcerative colitis (UC). Interestingly, there is known to be a degree of co-occurrence between these conditions (Egeberg *et al.*, 2016). Psoriasis, CD and UC share a number of genetic associations including interleukin family genes *IL12B* and *IL23R*, involved in cytokine-mediated immune response; *STAT3*, which is activated by the interleukin *IL6* (also shared) to produce inflammatory T-cells (Yang *et al.*, 2007); and (in psoriasis and UC) human leukocyte antigen *HLA-B*, which also plays an important role in the immune system. Psoriasis, CD and UC also show shared dysregulation in the expression of several genes including upregulation in the pro-inflammatory S100 family (*S100A8*, *S100A9*) and CXC chemokines *CXCL8*, *CXCL9* and *CXCL10* (associated with immune system activation). Importantly, some of their shared features are relevant to the drugs prescribed for these diseases: the monoclonal antibodies adalimumab and infliximab are antagonists of TNF (Park and Jeen 2015), whose corresponding gene variation in a number of diseases including CD, UC and psoriasis.

### 3.3 Similarity conversion allows comparison of information content between feature spaces

**Fig. 4.**
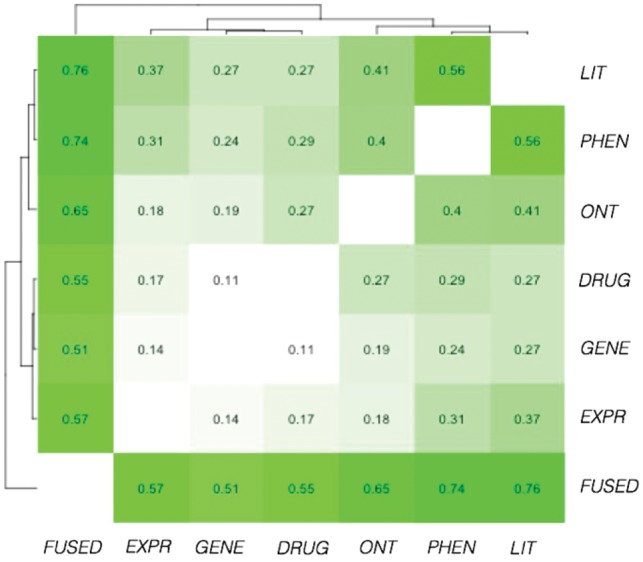
Correlation of pairwise similarity scores between feature spaces. The high correlation between phenotypic-, ontological- and literature-based similarity indicates that relationships in these ‘traditional’ spaces are relatively similar to each other, whereas there is little resemblance between relationships in genetic association, gene expression and drug spaces. The fused space resembles relationships in all spaces, but appears more similar to the ‘traditional’ spaces due to the multiple representation of relationships shared between these spaces

The use of quantile normalization allows the direct comparison of disease relationships present in the individual (and fused) feature spaces. This can be quantified by the Pearson correlation between the pairwise disease similarities in each space (Fig. 4). The most similar spaces are phenotype and literature co-occurrence, with a Pearson correlation of 0.56. Both spaces are based on literature mining, and there is also a degree of overlap between MeSH disease terms and phenotypes (e.g. ‘diabetes mellitus’ is both a MeSH disease term and a phenotype in the Human Phenotype Ontology) so the two spaces are not completely orthogonal. The ontological space also has high correlation with these two spaces, suggesting that these spaces capture ‘traditional’ knowledge of disease relationships. In contrast, the low correlation (<0.2) across the three ‘non-traditional’ representations (genetic association, gene expression and drug approval) indicate that disease relationships are highly distinct in each of these spaces.

Whilst the fused similarities have high correlation with each of the individual spaces, the fused space seems to resemble the three ‘traditional’ spaces more than the others, despite each space contributing equally to the fused similarities. As may be anticipated, shared similarities in the ‘traditional’ spaces cause the averaged similarities in the fused space to reflect these shared similarities more highly. This can be adjusted by down-weighting these spaces so that they have less influence on the fused similarities. Weighting the ‘traditional’ spaces so that they together make up one-third of the total similarity (instead of half), the similarity of the ‘traditional’ spaces to the fused becomes 0.56, 0.65 and 0.68 for ontological, phenotypic and literature-based spaces, respectively; and 0.58, 0.63 and 0.61 for genetic, expression and drug spaces. Despite doubling the contribution of the ‘non-traditional’ spaces, the resulting disease map does not appear substantially different (Supplementary Table S4, Supplementary Figs S5 and S6), suggesting that the disease map is not overly affected by the similarity of the ‘traditional’ spaces.

Similar results are seen for correlation of each individual space to only the significant disease similarities included in the disease map, rather than the entire fused matrix (i.e. setting non-significant fused similarities to 0) (Supplementary Table S5). The disease map therefore fundamentally resembles these traditional spaces, whilst inclusion of the diverse relationships from the genetic association, gene expression and drug spaces adds novel similarities which distinguish the disease map from traditional classification systems.

### 3.4 Top disease links in the fused space show high overlap in shared drugs relative to the individual spaces

One aim of the disease map is the identification of similarities between diseases that could indicate where two diseases might be treated with the same drug. We therefore assessed the extent to which links in the disease map correspond to drug-sharing relationships between the linked diseases. For this task, we included in the definition of drug-sharing drugs that are in phase 3 clinical trials (as opposed to approved drugs only, which were used to construct the drug feature space). 61.6% of the links in the full disease map share drugs (with 44.2% of links sharing approved drugs only).

Rather than simply looking at the percentage of links which share at least one drug, we can evaluate the mean Jaccard drug overlap of diseases linked by the map. This accounts for differences in the number of drugs prescribed for each disease, as well as the number of drugs shared. However, this score is less intuitive and is best understood in comparison with the individual disease maps. Excluding any information from drug space, we therefore compared the remaining individual spaces to a disease map constructed from the fusion of these five spaces. At the cut-off of the top 6.9% of similarity values (used to construct the full disease map), links in this non-drug fused space have a higher Jaccard overlap of drugs approved and in Phase III trials (0.050) than in any of the individual spaces (mean of 0.040).

**Fig. 5.**
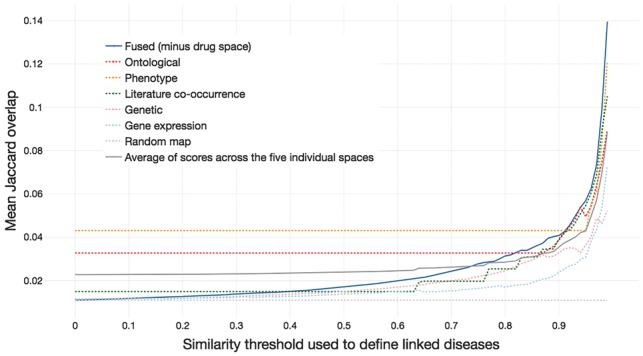
Mean Jaccard overlap of drugs between disease pairs linked at different thresholds of similarity. The mean Jaccard overlap of drugs (approved or in Phase III clinical trials) was measured at 100 similarity thresholds, from 0.00 to 0.99. Diseases that are highly similar in the fused space (constructed without drug information) are more likely to share approved or trialled drugs than diseases that are highly similar in the five individual spaces on average. Drug overlap in the sparse feature spaces, which have comparably few links between diseases, is static until higher thresholds of similarity are used (noticeable for the ontological and phenotypic spaces)

This analysis was repeated across multiple similarity thresholds, from all values to the top 1% highest similarity scores (Fig. 5). As expected, the higher the similarity threshold used, the greater the mean Jaccard drug score of diseases linked in the resulting map. Indeed, at the top thresholds of similarity (the top 5% or above), links in the non-drug fused map show greater mean drug overlap than links in any of the maps constructed from individual spaces, although the difference is relatively small. Importantly, drug overlap at the top thresholds is higher for the fused similarities than the average over the five spaces (grey line on Fig. 5), despite the fact that the fused similarities are constructed from the average of similarities in each space. A similar result was also seen when considering only approved drugs (Supplementary Fig. S7) and for the weighted disease map (Supplementary Fig. S6), although for these cases ontological and/or literature spaces slightly outperform the non-drug fused space at certain (including top) similarity thresholds. If only novel links (those in different top-level DO classes) are considered, the fused space is outperformed by ontological and literature spaces; this may be driven by the presence of links between neurodegenerative and mental disorders in these spaces (see Supplementary Fig. S8 for details).

### 3.5 Fused similarities outperform individual similarities in the prediction of disease classes

**Fig. 6.**
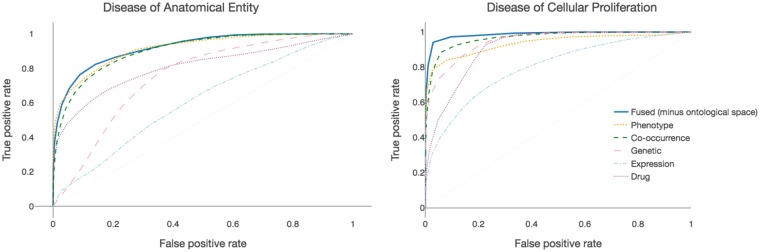
Ability of similarity scores from fused and individual spaces to predict Disease Ontology classes. Individual spaces differ widely in their predictive ability, with literature-based similarity and phenotypic similarity performing particularly well. The fused similarity scores outperform all individual spaces for the prediction of ‘disease of cellular proliferation’ (AUROC 0.986, right). The fused similarity scores also outperform the individual spaces for predicting ‘disease of anatomical entity’ (AUROC 0.920), although for this class (which contains more diverse disease types) phenotype and literature co-occurrence perform almost as well (AUROC 0.901 and 0.905, respectively)

We next used Random Forest classifiers to examine how well the similarities in fused and individual spaces (excluding the ontological space) correspond to known disease categories, reasoning that the ability of the fused similarities to reconstruct known categories would grant greater confidence that any novel relationships are likely to be biologically relevant. To ensure the existence of sufficient training data to build a robust classifier, we aimed to predict the two largest Disease Ontology classes: *disease of anatomical entity* and *disease of cellular proliferation*. Receiver Operating Characteristic curves for each space show that there is high variation between each space, although all spaces did better than random (Fig. 6). Of the individual spaces, literature-based similarities were best able to classify diseases into known categories, with an AUROC of 0.905 for *disease of anatomical entity* and 0.968 for *disease of cellular proliferation*. Phenotypic similarities were also good predictors of disease classes, with an AUROC of 0.901 and 0.927 for *disease of anatomical entity* and *disease of cellular proliferation*, respectively. Genetic and transcriptomic spaces do not closely correlate with the known categorizations (Fig. 6), which is expected as traditional disease classifications (such as the slightly outperform the non-drug fused space at certain (including top) similarity thresholds) do not take into account genetic or transcriptomic similarities.

The (non-DO) fused similarities outperformed any of the individual spaces, with AUROC scores of 0.920 for *disease of anatomical entity* and 0.986 for *disease of cellular proliferation*, despite the integration of spaces which are not such good predictors of disease classes. The mean performance over the five individual spaces was 0.795 for the prediction of *disease of anatomical entity* and 0.910 for the prediction of *disease of cellular proliferation*, meaning that the non-DO fused similarities outperformed the individual similarities by 10% on average (mean AUROC over the two tasks of 0.953 for the fused similarities versus 0.852 for the individual similarities). Largely similar results were seen for different feature sizes (Supplementary Fig. S2), although phenotypic and/or literature spaces slightly outperformed the fused space at some feature set sizes. Weighting the fused similarities so that the overlapping phenotype and literature co-occurrence spaces accounted for only 25% of the fused similarities (instead of 40%, as ontological space is excluded) did not significantly affect classification of *disease of cellular entity*, but slightly reduced the AUROC score to 0.891 for *disease of anatomical entity* (slightly less than phenotypic and literature similarities, see Supplementary Table S4).

## 4 Discussion

In this study, we have introduced a method to integrate biological data across multiple domains through conversion of feature sets into normalized similarity scores, such that each space contributes evenly to the fused similarity. For the first time, we applied this similarity fusion approach across six feature spaces (ontological, phenotypic, literature co-occurrence, genetic association, gene expression and drug indication data) in an unbiased manner, avoiding the need to make any judgements as to the importance of different data types. Following the normalization step relative, spaces may be weighted according to the desired application of the map (in terms of the importance placed on finding novel links versus reflecting known links, for instance). Given that this choice is application-dependent, we here used a balanced fusion of each space to create a ‘disease map’: a network linking diseases with significant similarities across multiple spaces. As well as known disease connections involving clinically related and comorbid diseases, the disease map reveals novel connections between diseases in different ontological categories (Fig. 2), and highlights shared features between diseases—for example, shared gene expression patterns which may underlie an observed common phenotype. In the case study of psoriasis, we showed how genetic variants shared with inflammatory bowel diseases were also related to drugs used for both conditions, illustrating how the identification of similarities between diseases at a ‘molecular’ level can indicate potential opportunities for sharing drugs, and to generate early drug-repositioning hypotheses in a ‘guilt-by-association’ approach (Chiang and Butte 2009). This example illustrates how ‘molecular’ (e.g. genetic and gene expression based) approaches to disease similarity can identify disease relationships which are not captured by traditional classifications of disease: the link between psoriasis and autoimmune disease, for example, is present in SNOMED but absent from other major classifications including MeSH, DO and ICD.

Through the fusion of multiple data types, the disease map gives a new perspective on disease relationships, where aspects of disease not ordinarily considered by established classification systems (such as genetics and gene expression) reveal novel similarities between diseases. These spaces contain similarities not captured in our ‘traditional’ understanding of disease relationships (Fig. 4), and therefore contribute greater depth of interest to the disease map.

From this perspective, the more data types that can be included in the map, the more complete the description of the biological system becomes. Using a ‘hold-out’ evaluation style, we were able to incorporate all six data types included in this study into the map, without designating any data types as reserved for evaluation purposes. In agreement with previous studies showing how inclusion of more data types leads to greater accuracy in the prediction of disease relationships (Sun *et al.*, 2014a; Žitnik *et al.*, 2013), the fused similarities outperformed any individual space in predicting disease class membership, despite the inclusion of spaces that individually had little relation to known disease classes (Fig. 6). In fact, the fused similarities, which are based on averaging similarity values, outperformed the average of individual similarity values by a mean of 10% across the two classes. One explanation for this is that the two spaces that are most similar to the ontological space (phenotypic and literature co-occurrence spaces) are also similar to each other (Fig. 4), as they are based on literature mining of phenotype terms and MeSH terms respectively, and there is some overlap between these term sets. The similar disease relationships contained in these spaces therefore reinforce each other in the fused similarities. However, even if these two spaces are down-weighted (with a corresponding increase in influence of the ‘non-traditional’ spaces), the fused similarities still markedly outperform the average of other spaces in the prediction of disease classes (Supplementary Table S4), suggesting that the classification performance is not driven purely by these spaces; rather, the benefit in similarity fusion lies in the prioritization of disease relationships common to multiple spaces.

Our second evaluation measure was the sharing of drugs (either approved or in Phase 3 clinical trials) between diseases. Although the drug sharing space is highly distinct from any of the other spaces (Fig. 4), drug-sharing relationships were captured well by the (non-drug) fused space, which had a high mean Jaccard overlap of drugs shared amongst its most similar disease pairs relative to the individual spaces (Fig. 5). This not only increases our confidence in the biological relevance of the linked diseases, it further illustrates the value of incorporating multiple data types into the disease map. This pattern fits what we have seen in computational drug-repurposing approaches: while approaches based on individual data types such as genome-wide association studies (Okada *et al.*, 2014) or transcriptomics (Dudley *et al.*, 2011; Jahchan *et al.*, 2013; van Noort *et al.*, 2014) are possible, successful drug-repurposing methods often incorporate multiple data types (Gottlieb *et al.*, 2011; Iwata *et al.*, 2014); data fusion may therefore become an increasingly important approach in drug discovery.

In summary, we have demonstrated the utility of similarity fusion for integrating different types of biological data in the analysis of disease relationships, showing that the fused data is not only able to reconstruct known disease and drug-sharing associations, but also offers the possibility of highlighting new relationships between diseases. Our similarity-based approach is particularly suited for the integration of high-throughput datasets where dimensionality would otherwise pose a problem, such as proteomics and metabolomics data. This approach could be extended to any number of spaces, leading to the possibility of a fully comprehensive disease map. Such a map could transform our current understanding of disease and disease relationships, revealing shared mechanisms behind diverse diseases which could eventually help to drive novel drug repurposing and treatment opportunities.

## Supplementary Material

Supplementary Data
