# Supplementary material for: Understanding and predicting disease relationships through similarity fusion
Source: Bioinformatics. 2018 Aug 30;35(7):1213–20. doi: 10.1093/bioinformatics/bty754 (PMC6449746; doi:10.1093/bioinformatics/bty754)
Supplement: Supplementary Data [file bty754_supp.zip › bty754-Suppl_data/bty754_Supplementary_Data.docx]

**Supplementary Text 1: Creation of feature vectors for each feature space**

Although we aim for 100 features for each disease in each space, the exact number of features may vary slightly depending on the available data, e.g. due to fewer than 100 genes or co-occurring terms being annotated to a disease, duplicated phenotypes, or ties for the 100th similarity value.

**Ontological feature space**: The Disease Ontology^1^ was downloaded from <http://ontologies.berkeleybop.org/doid.obo> in December 2016 and used to match disease names to their Disease Ontology ID, which is required for the following step. The DOSE package^2^ was used to measure semantic similarity between terms using Lin’s measure^3^, which minimizes the number of ties between disease terms. The feature set for each disease was then calculated as the top 100 most similar diseases according to this metric, excluding self-similarity. When there are ties for the top 100^th^ similarity value, all diseases are retained, so some diseases have more than 100 features, up to a maximum of 135. Two diseases (irritable bowel syndrome and polycystic ovary syndrome) have no similar diseases according to DOSE, so these are not assigned any features.

**Phenotypic feature space**: Disease-phenotype associations were taken from the work of Hoehndorf et al.^4^ who matched diseases with terms from the Human and Mammalian Phenotype Ontologies based on literature co-occurrence. This dataset, which comprises the 21 most highly-associated Human/Mammalian Phenotype Ontology terms for each disease, was obtained from <http://aber-owl.net/aber-owl/diseasephenotypes/data/> in November 2016, and this formed the feature set for the phenotypic feature space. There are some duplicated phenotypes in the supplied data, so there are only 19 or 20 phenotypes for 16 of the 84 diseases.

**Literature co-occurrence feature space***:* 13 million Medline abstracts (dating between 2000 and 2016) were annotated with MeSH term identifiers using a recently published named entity recognition system, TaggerOne^5^. These terms mostly represent MeSH disease concepts, although they also include a few more general concepts such as ‘body weight’ or ‘infection’, or higher level disease terms such as ‘nervous system diseases’ or ‘musculoskeletal abnormalities’. The feature set for this space was the top 100 most highly co-occurring MeSH terms according to the normalized pointwise mutual information (NPMI) score, excluding self-similarity. Three diseases had less than 100 co-occurring terms (allergic contact dermatitis with 23, male infertility with 55, and juvenile rheumatoid arthritis with 80). Certain MeSH terms overlap with the Human/Mammalian Phenotype Ontology terms used for the phenotypic space (e.g. ‘diabetes mellitus’, ‘neoplasms’ and ‘carcinoma’ are examples of terms that are included in both sets), so there is a degree of overlap between these two spaces.

**Genetic feature space***:* Disease-gene associations were downloaded from DisGeNET^6^ ([http://www.disgenet.org/web/DisGeNET/menu/downloads](http://www.disgenet.org/web/DisGeNET/menu/downloads))) in November 2015. These mostly represent associations of type ‘genetic variation’, which includes susceptibility mutations, causal mutations, and modifying mutations; there are also a small number of associations of type ‘post-translational modification’ and ‘therapeutic’. Associations of type ‘AlteredExpression’ were removed to avoid overlap with the transcriptomic feature space, and entries for the non-gene ‘NEWENTRY’ were removed. The feature set was composed of the top 100 associated genes by evidence score. There was high variation in the number of genes associated to each disease: 19 diseases have less than 100 associated genes (10 diseases have less than 20), but there were also a large number of ties in the data due to the calculation of the evidence score for each gene, leading some diseases to have more than 100 associated genes. 10 diseases had more than 100 genes, up to a maximum of 207 genes for malignant pleural mesothelioma.

**Transcriptomic feature space**: Gene expression microarray experiments were selected as described in the main text, and analyzed in R version 3.3.2. CEL files downloaded from GEO were RMA-normalized using the Affy package^7^ and annotated using code adapted from the web tool GEO2R^8^. Differential expression profiles for each probe were generated using the limma package, version 3.30.13^9^, and mapped to gene-level retaining the probe with the highest statistical significance (lowest p-value) in the case of a gene mapping to multiple probes, and retaining all genes in the case of a probe mapping to multiple genes. The feature ‘universe’ was defined as the set of 4482 genes measured in all experiments. The feature set for each disease was calculated as the top 100 of these genes by absolute log-fold change at a p-value threshold of <0.05. No multiple testing correction was used, in order to ensure that all datasets had at least 100 differentially expressed genes. This list was then split into separate feature sets for up- and down-regulation.

**Drug feature space**: Drug indication data was downloaded from ChEMBL version 22.1^10^ (<https://www.ebi.ac.uk/chembl/downloads>). The feature set comprised approved drugs for each condition. The number of approved drugs listed for each disease in ChEMBL ranges from 0 (for 11 conditions, including four diseases – dengue fever, leukopenia, limb-girdle muscular dystrophy, and measles – which could not be mapped to EFO or MeSH terms used by ChEMBL) to 72 (for type II diabetes).

The spaces have different sparsities: phenotypic space is the most sparse, with only 7.5% of disease pairs having any overlap in their phenotypes; followed by ontological at 12.9%, drug at 13.6%, co-occurrence at 58.5%, genetic at 83.3%, and finally transcriptomic space, with 84.4% of disease pairs having some overlap. This is related to the size of the feature set in each space relative to the size of the feature universe.

**Independent comorbidity dataset**

Comorbidity associations based on Medicare records of 13 million patients^11^ were downloaded from [sbi.imim.es/data/hudine](http://sbi.imim.es/data/hudine). Diseases are recorded in this data as ICD9 3-digit codes; mapping our diseases to these codes resulted in duplicated codes for 14 diseases (e.g. type 1 and type 2 diabetes mellitus both map to 250 *diabetes mellitus*; bipolar disorder and major depressive disorder both map to 296 *episodic mood disorders*; see Supplementary File 2 for mappings).

Disease pairs with less than 100 co-occurrences were filtered out (as the relative risk (RR) comorbidity measure tends to overestimate for pairs with small numbers of observed associations^12^), leaving 88,347 disease pairs for which comorbidity data was recorded. 800 of these observations related to disease pairs in our dataset, which when the 14 duplicate diseases are included covers 938 (27%) of our 3486 disease pairs. Relative risk (RR) was used to quantify comorbidity, where a RR of 1 indicates that diseases occur together as often as expected by chance. The lower quantile, median, and upper quantile values of RR in the 800 recorded pairs were 0.76, 1.07, and 1.62.

RR thresholds of 1.5, 2, and 5 were used to define comorbid disease pairs, with 239, 125 and 32 of the 800 observed disease pairs respectively meeting these thresholds. Of our 938 disease pairs for which comorbidity data exists, we compared the percentage of the 63 disease pairs linked in the map vs the 875 disease pairs not linked in the map which were comorbid at these thresholds. In both cases, duplicate pairs were counted twice (e.g. “type 1 diabetes-obesity” and “type-2 diabetes-obesity” were counted as two separate pairs although they both map to “250-278” at the ICD code level).

**Supplementary Text 2**

For the phenotypic space, the feature set size was limited to the 21 phenotypes supplied in the Hoehndorf dataset; for drug space, the feature set size was limited by the number of drugs indicated for each disease, and so different feature set sizes could not be tested for these spaces. We chose to use a feature set size of 100 for the remaining spaces, which captures sufficient information in each space whilst not being overly large compared to the fixed-size feature spaces. To test the dependence of the results on the chosen feature set size, different feature set sizes of 20, 50, and 200 were tested were tested for ontological, literature co-occurrence, genetic, and transcriptomic feature spaces.

As expected, the proportion of links that were significantly greater than those observed in random maps (and therefore the number of links in each map) varied depending on the feature set size, with larger feature set sizes resulting in greater differentiation from random maps (Supplementary Table 1). Related to this, the mean Jaccard overlap of drugs shared by diseases linked in the map increases at smaller feature set sizes (because at smaller feature set sizes only the highest similarity links pass the random significance threshold and are included in the map).

Evaluating the full similarity matrices (in terms of the proportion of links which share drugs, and the ability to predict DO categories) produced similar results at different feature set sizes, with some minor variations in the performance of individual feature spaces (Supplementary Figures 1 and 2). Finally, at all feature set sizes, literature co-occurrence, phenotype, and ontological spaces were the most highly correlated to the fused space.

**Supplementary Table 1. Comparison of disease maps at different feature set sizes**

|  | Feature set size | | | |
| --- | --- | --- | --- | --- |
|  | 20 | 50 | 100 | 200 |
| Percentage of links in full similarity matrix classed as significant (and therefore included in the resulting disease map) | 2.98 | 4.76 | 6.91 | 9.12 |
| Percentage of links in disease map classed as novel | 16.2 | 15.6 | 15.3 | 16.3 |
| Mean Jaccard overlap of drugs shared by diseases linked in the disease map (approved or in Phase III trials) | 0.099 | 0.082 | 0.069 | 0.061 |
| Mean Jaccard overlap of drugs shared by diseases linked in the disease map (approved drugs only) | 0.107 | 0.081 | 0.069 | 0.059 |


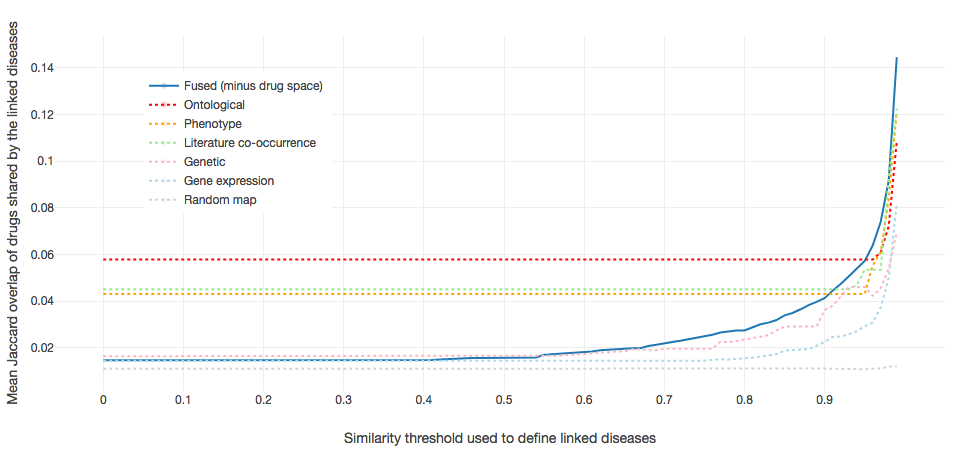

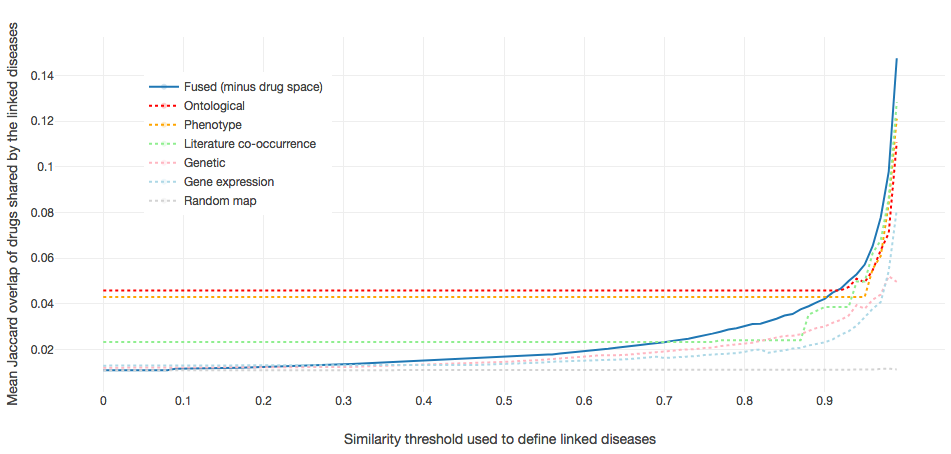


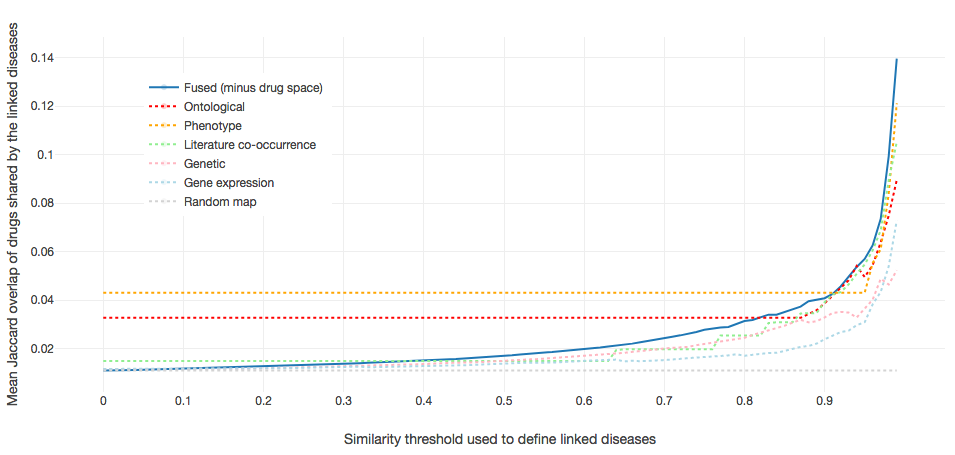

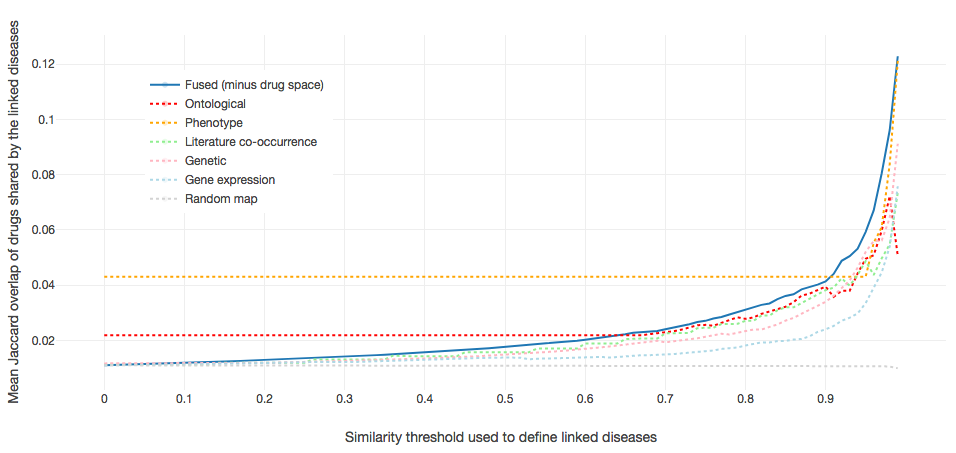


**Supplementary Figure 1. Comparing the mean Jaccard drug overlap (approved or in Phase III clinical trials) of links in disease networks at different thresholds of similarity, and different feature vector sizes.** Similar overall results are obtained for feature vector sizes of 20 (top left), 50 (top right), 100 (bottom left), and 200 (bottom right), with some minor variation in the performance of individual spaces. In particular, at a feature vector size of 20, literature space and ontological space marginally outperform the fused space at certain thresholds. The effect of different feature vector sizes on sparsity can be observed here, particularly for the ontological space, which shows higher sparsity at smaller feature set sizes. This indicates that smaller feature sets are insufficient to capture much overlap between diseases in this space (compared to the size of the feature universe).


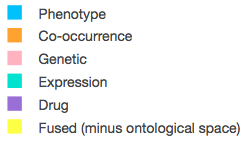


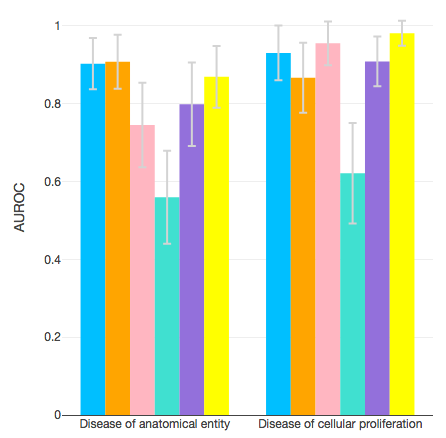

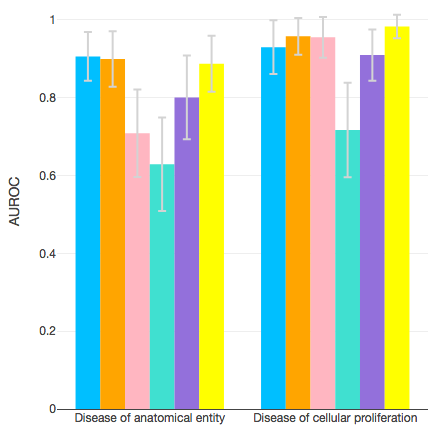

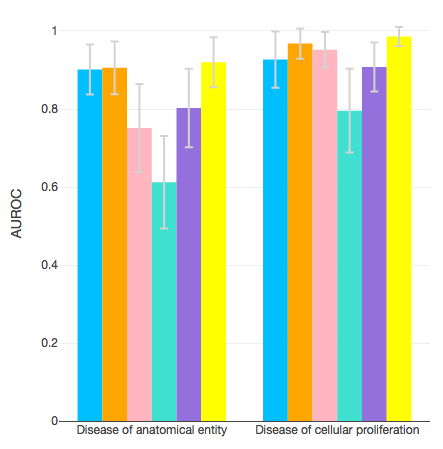

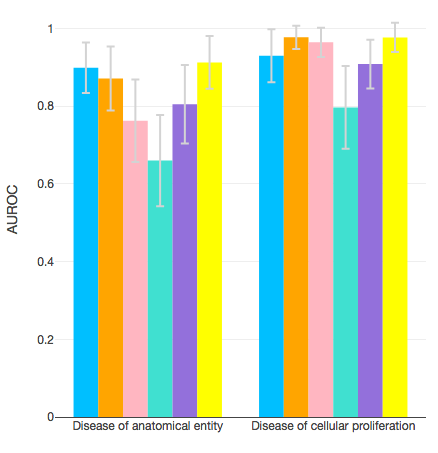


**Supplementary Figure 2. Random Forest AUROC scores for individual and fused disease similarities at different feature vector sizes.** Overall, results are similar at feature vector sizes of 20 (top left), 50 (top right), 100 (bottom left), and 200 (bottom right) , and improve with increases in feature vector size. For the class *disease of anatomical entity*, AUROC scores for the fused disease map (minus the ontological space) improve from 0.869 for length 20, to 0.913 for length 200, but literature co-occurrence and phenotypic spaces outperform the fused space at feature set sizes 20 and 50. For the class *disease of cellular proliferation*, the fused map outperforms any of the individual maps at feature set sizes of 20, 50, and 100*;* at a feature set size of 200 it performs equally to literature co-occurrence (with AUROC scores of 0.9769 and 0.9768 respectively).

**Supplementary Text 3: Example of fused similarity calculation between lung cancer and pancreatic cancer**

For simplicity, only two spaces will be used in this example: phenotypic and drug space.

ChEMBL (version 22.1) lists 15 approved drugs for non-small-cell lung carcinoma, and 3 for pancreatic cancer; of these, they share the chemotherapeutic gemcitabine hydrochloride. This gives them a Jaccard similarity score of 0.059 (this is the size of the intersect of the feature sets divided by the size of the union of the feature sets: 1/17).

Of the 21 literature-mined phenotypes for each disease, three are shared ("adenocarcinoma, glandular cancer, glandular carcinoma, increased adenocarcinoma incidence"; "increased neoplasm incidence, increased tumor incidence, spontaneous tumor"; and "tumorigenesis") giving them a Jaccard similarity score of 0.077 in phenotype space.

Quantile normalization involves replacing each value with the mean value of the same rank across each space. In this example, the maximum similarity scores are 0.556 in phenotypic space and 1 in drug space, and so the maximum similarity score in both spaces is replaced with the mean of these two values: 0.778. The second highest values are 0.481 in phenotypic space and 0.500 in drug space, so these scores are replaced in each space with 0.4905, and so on. Adjustment for ties is used, so that tied ranks are replaced with the mean quantile normalized value across those ranks (see orange values in Supplementary Table 2).

**Supplementary Table 2. Example of quantile normalization with adjustment for ties**

Where there are ties for a particular rank in one space, the values are replaced by the mean of the quantile-normalized values for those ranks: here the tied values in phenotypic space are replaced by the mean of 0.475, 0.470, and 0.465 = 0.470.

|  | Raw similarity scores, drug space | Raw similarity scores, phenotypic space | New value in drug space after quantile normalization | New value in phenotypic space after quantile normalization |
| --- | --- | --- | --- | --- |
| Highest value | 1 | 0.556 | 0.778 | 0.778 |
| 2^nd^ highest value | 0.500 | 0.481 | 0.491 | 0.491 |
| 3^rd^ highest value | 0.490 | 0.460 | 0.475 | 0.470 |
| … | 0.480 | 0.460 | 0.470 | 0.470 |
| … | 0.470 | 0.460 | 0.465 | 0.470 |


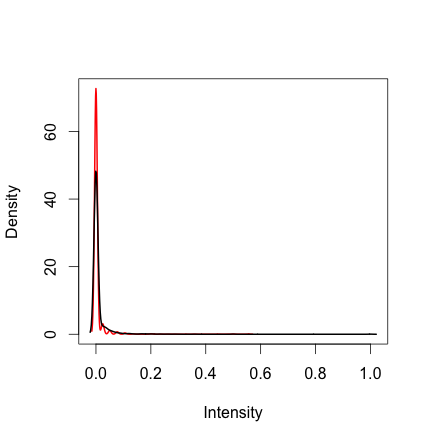

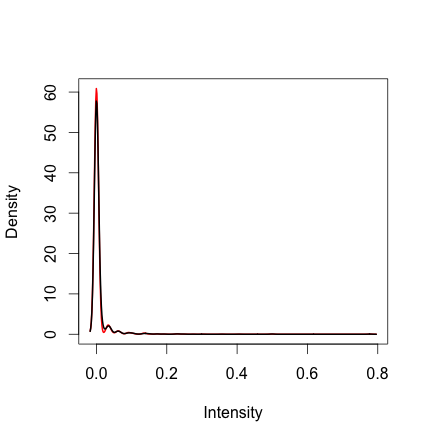


**Supplementary Figure 3. Distribution of similarity scores in phenotypic and drug space before (left) and after (right) quantile normalization.**

Red line: phenotypic space; black line: drug indication space.

Before quantile normalization of the 3486 pairwise similarity scores from each space, the similarity scores range from 0 to 0.556 in phenotypic space, with a sum of 16.9, and from 0 to 1 in drug space with a sum of 29.9. Following quantile normalization, the similarity scores in both spaces range from 0 to 0.778, and the sum of similarities was 21.0 and 23.4 in phenotypic and drug space respectively (due to the presence of ties, the two distributions are not perfectly equal, as seen in Supplementary Figure 3). The quantile-normalized similarity scores of the two diseases are 0.091 in phenotypic space and 0.042 in drug space. The fused similarity score of lung cancer and pancreatic cancer is then simply the mean of these two scores: 0.067.


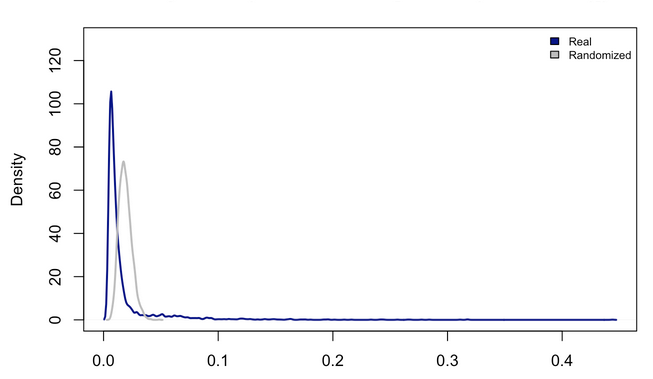


**Supplementary Figure 4. Distribution of the fused similarity scores vs fused scores created from randomly sampled feature vectors in each space.** This figure shows an example for one of the 1000 random matrices created. On average, the real scores show less overlap between diseases than random; however, the distribution of real scores has a long tail with 8.5% of values being greater than the maximum similarity value of 0.05 observed in this particular set of random scores (by comparison, the maximum similarity score observed in the real similarities is 0.44).

| Novel link | | Number of shared: | | | | |
| --- | --- | --- | --- | --- | --- | --- |
|  |  | Phenotypes | MeSH terms co-occurring in literature | Genetic associations | Dysregulated genes | Drugs |
| Acne | Actinic keratosis | 1 | 15 | 1 | 18 | 0 |
| Acne | Polycystic ovary syndrome | 1 | 16 | 0 | 15 | 0 |
| Actinic keratosis | Atopic dermatitis | 0 | 10 | 0 | 23 | 0 |
| Actinic keratosis | Psoriasis | 0 | 15 | 6 | 25 | 0 |
| Actinic keratosis | Rosacea | 3 | 15 | 4 | 13 | 0 |
| Alcoholism | Head and neck squamous cell carcinoma | 0 | 33 | 2 | 2 | 0 |
| Alzheimer's disease | Down syndrome | 0 | 4 | 17 | 3 | 2 |
| Bacterial meningitis | Influenza | 1 | 12 | 4 | 35 | 1 |
| Cervical squamous cell carcinoma | Chronic hepatitis b (carrier) | 0 | 6 | 42 | 0 | 0 |
| Cervical squamous cell carcinoma | Chronic hepatitis c | 0 | 5 | 41 | 1 | 0 |
| Cervical intraepithelial neoplasia | Dengue fever | 0 | 0 | 5 | 29 | 0 |
| Chronic obstructive pulmonary disease | Non-small cell lung carcinoma | 2 | 4 | 1 | 20 | 0 |
| Chronic obstructive pulmonary disease | Malignant pleural mesothelioma | 2 | 2 | 2 | 19 | 0 |
| Colorectal adenocarcinoma | Crohn's disease | 3 | 34 | 12 | 21 | 0 |
| Colorectal adenocarcinoma | Irritable bowel syndrome | 0 | 29 | 9 | 7 | 0 |
| Colorectal adenocarcinoma | Ulcerative colitis | 3 | 35 | 16 | 26 | 0 |
| Crohn's disease | Dengue fever | 5 | 1 | 17 | 4 | 0 |
| Crohn's disease | Irritable bowel syndrome | 0 | 22 | 27 | 6 | 4 |
| Dengue fever | Systemic lupus erythematosus | 0 | 10 | 15 | 22 | 0 |
| Dengue fever | Ulcerative colitis | 5 | 2 | 15 | 3 | 0 |
| Dengue fever | Vulvar intraepithelial neoplasia | 0 | 0 | 0 | 24 | 0 |
| Down syndrome | Huntington's disease | 0 | 4 | 12 | 0 | 1 |
| Endometrial carcinoma | Endometriosis | 7 | 21 | 8 | 1 | 0 |
| Endometriosis | Polycystic ovary syndrome | 3 | 12 | 7 | 1 | 0 |
| Endometriosis | Prostate cancer | 3 | 3 | 6 | 1 | 1 |
| Chronic hepatitis b (carrier) | Hepatocellular carcinoma | 3 | 29 | 0 | 2 | 2 |
| Chronic hepatitis b (carrier) | Sarcoidosis | 0 | 2 | 35 | 9 | 1 |
| Chronic hepatitis c | Hepatocellular carcinoma | 3 | 28 | 0 | 0 | 0 |
| Chronic hepatitis c | Sarcoidosis | 0 | 2 | 31 | 15 | 0 |
| Idiopathic pulmonary fibrosis | Non-small cell lung carcinoma | 2 | 6 | 7 | 19 | 0 |
| Influenza | Leukopenia | 2 | 5 | 9 | 21 | 0 |
| Influenza | Sarcoidosis | 0 | 1 | 16 | 21 | 0 |
| Irritable bowel syndrome | Ulcerative colitis | 0 | 24 | 27 | 8 | 1 |
| Myocardial infarction | Type 1 diabetes mellitus | 0 | 0 | 17 | 26 | 2 |
| Obesity | Polycystic ovary syndrome | 0 | 27 | 4 | 1 | 3 |
| Sarcoidosis | Type 1 diabetes mellitus | 0 | 2 | 26 | 15 | 0 |
| Sickle cell disease | Essential thrombocythemia | 0 | 10 | 14 | 5 | 1 |

**Supplementary Table 3. Novel links between diseases in different Disease Ontology classes.** Novel links are disease pairs which have similarity higher than the significance threshold, but which are not related by the Disease Ontology top-level classes. Many novel links are related in multiple feature spaces, indicating similarities on different biological levels. Some relationships which fall under this definition are expected, such as the connection between inflammatory bowel disease (DO class ‘disease of anatomical entity) and colorectal cancer (DO class ‘disease of cellular proliferation’), as they affect the same organ system. Other connections, such as between hepatitis B and cervical cancer, seem surprising, and in such cases it is helpful to interpret the features shared between the two diseases, such as the 42 shared genetic associations. These include genes in the human leukocyte antigen system (*HLA-A, HLA-B, HLA-C, HLA-DPB1, HLA-DQA1, HLA-DQB1,* and *HLA-DRB1*), which are involved in antigen presentation; this suggests that the link between the two diseases is driven by shared aspects of immunological response. This may reflect the involvement of the human papillomavirus (HPV) in the majority of cervical cancer cases, with the immune response playing a key role in the development of cervical cancer from an initial HPV infection^13^. Likewise, hepatitis B is a causal risk factor in the development of hepatocellular cancer^14^, so shared processes between the two diseases could also reflect the interface between infection and carcinogenesis of these two DNA viruses.


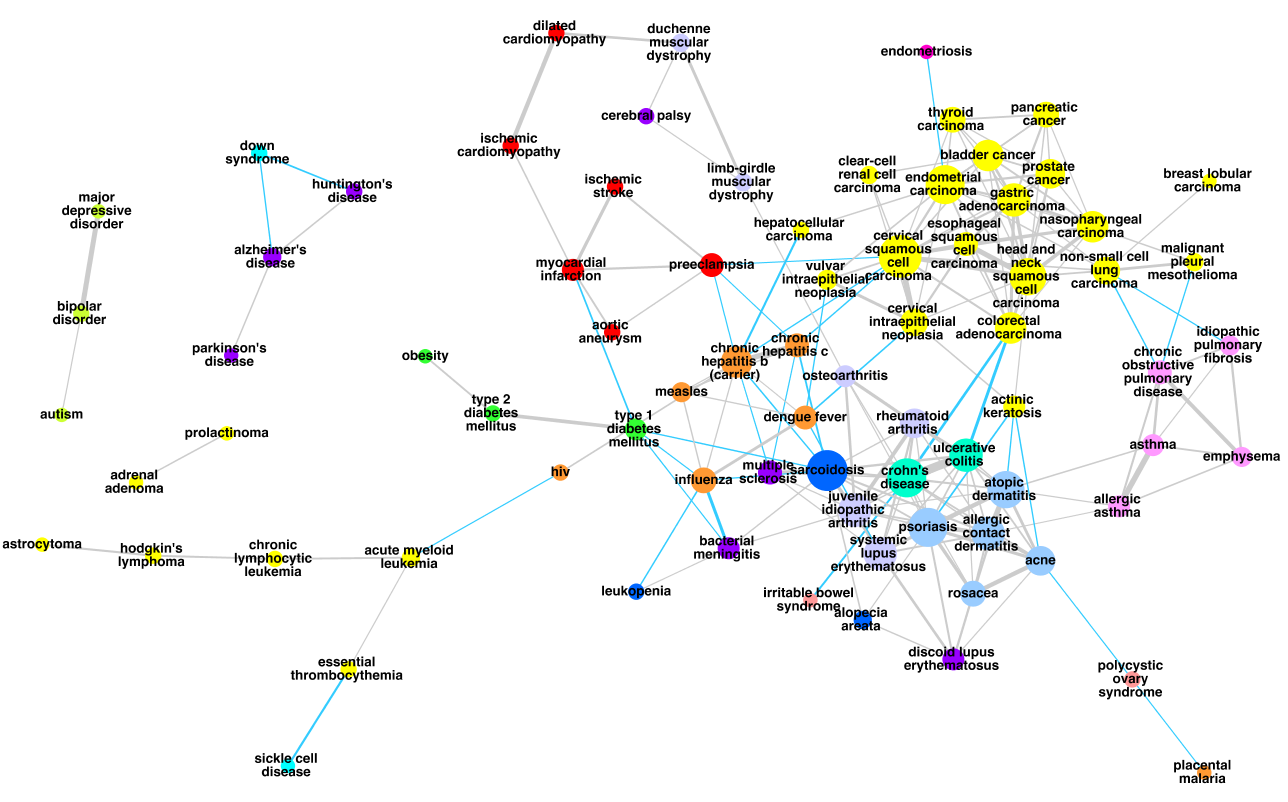


**Supplementary Figure 5. Disease map resulting from weighted similarity fusion**

The map shown here is constructed from a weighted mean of the individual spaces, so that the ‘traditional’ spaces (ontological, phenotypic, and literature co-occurrence similarity) together make up only a third (instead of a half) of the fused similarities (weighting scheme 1,1,1,2,2,2). The resulting disease map looks similar to the balanced (unweighted) disease map shown in Figure 2.

**Supplementary Table 4. Comparison of balanced and weighted fused similarities**

|  | Balanced | Weighted |
| --- | --- | --- |
| Percentage of values above significance threshold | 6.91 (242 links) | 6.02 (211 links) |
| Percentage of novel links in the disease map | 15.3 (37 links) | 17.1 (36 links) |
| Mean Jaccard overlap of drugs shared by diseases linked in the disease map, approved and Phase III/approved only | 0.069/0.069 | 0.075/0.077 |
| Mean Jaccard overlap of drugs shared by novel links in the disease map, approved and Phase III/approved only | 0.025/0.04 | 0.021/0.038 |
| AUC of DO class prediction, disease of anatomical entity/disease of cellular proliferation | 0.924/0.985 | 0.891/0.979 |

This table shows the results of adjusting the weights on the similarities so that the three ‘traditional’ spaces which show high similarity to each other (ontological, phenotype, and literature-based spaces) account for only a third (instead of half) of the fused similarities, i.e. the contribution of the other three spaces (genetic, expression, and transcriptomic) is doubled (weighting scheme 1, 1, 1, 2, 2, 2). Down-weighting these highly similar ‘traditional’ spaces means that they have less influence on the resulting disease map, however, the results shown here indicate that this down-weighting makes little difference to properties of the resulting disease map. For the DO class prediction, ontological space is excluded from the fused matrix, meaning that phenotypic and literature spaces together account for 25% of the fused similarities (weighting scheme 1, 1, 2, 2, 2). After this weighting, the fused space is no longer the best-performing space for the prediction of *disease of anatomical entity* (being slightly outperformed by phenotypic and literature co-occurrence similarities at 0.902 and 0.905 respectively). Note that the AUC quoted here for the balanced kernel varies slightly from that in the main text as the classifier was re-run.

Similar results are seen for different feature vector sizes, with the exception of the percentage of drug-sharing links which show slightly higher variation between the balanced and weighted maps particularly at smaller feature vector sizes (possibly due to the reduced number of links in the disease map at smaller sizes); however the overall mean Jaccard drug overlap of linked diseases (a more comprehensive measure which gives similarity across a whole range of thresholds) are similar to Supplementary Figure 6 across the different feature set sizes.


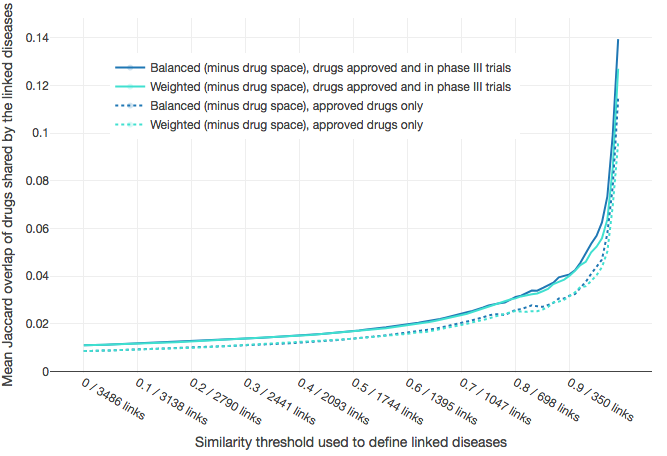


All links


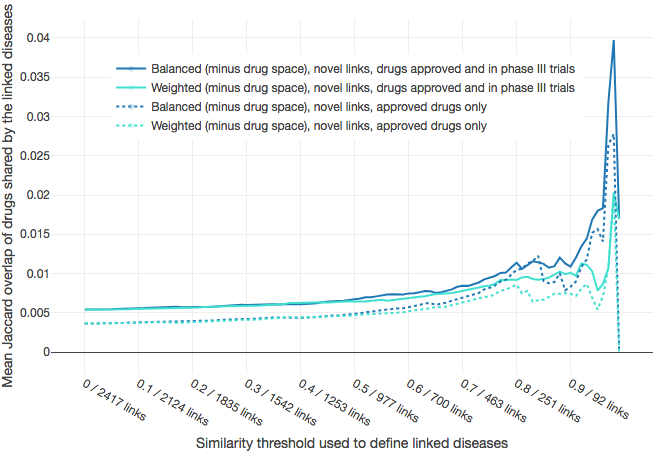


Novel links only

**Supplementary Figure 6. Comparing the drug overlap of linked diseases in weighted and balanced spaces**

In this case, drug space is excluded from the evaluation. The contribution of the two remaining non-traditional spaces is therefore tripled so that the traditional spaces contribute a third of the similarity to the weighted space (weighting scheme 1, 1, 1, 3, 3). The overlap of drugs for each link (mean Jaccard score) is very similar for balanced and weighted spaces. Note however that the weighted fused space is outperformed by literature space at the top 5% of similarities, in contrast to the balanced space which is the best performing space at the top 5% of similarities. As in Supplementary Table 4, the only major difference between the two spaces is seen if only novel links are considered (bottom plot), where this variation may be due to the small numbers of novel links between the two spaces.

**Supplementary Table 5. Pearson correlation of each individual space to the fused similarities**

The Pearson correlation of the pairwise disease similarities in the individual spaces against the fused space gives an indication of how much each space contributes to the fused similarities. Where the contribution of the ‘traditional’ spaces is down-weighted, a reduction is seen in their correlation to the fused matrix (with a corresponding increase in correlation from the genetic, transcriptomic, and drug spaces). We can also examine how much each space contributes to the significant similarities that make up the disease map, by computing the correlation against the significant similarities only (i.e., by setting all non-significant fused similarities, which are not included in the disease map, to 0). Patterns of correlation to the disease map are similar to those for the full fused similarities.

|  | Ontological space | Phenotypic space | Literature co-occurrence space | Genetic space | Transcriptomic space | Drug space |
| --- | --- | --- | --- | --- | --- | --- |
| Correlation to fused matrix (as shown in Fig. 4) | 0.65 | 0.74 | 0.76 | 0.51 | 0.57 | 0.55 |
| Correlation to fused weighted matrix | 0.56 | 0.65 | 0.68 | 0.58 | 0.63 | 0.61 |
| Correlation to disease map | 0.63 | 0.71 | 0.74 | 0.47 | 0.52 | 0.53 |
| Correlation to weighted disease map | 0.51 | 0.60 | 0.63 | 0.53 | 0.59 | 0.58 |

**
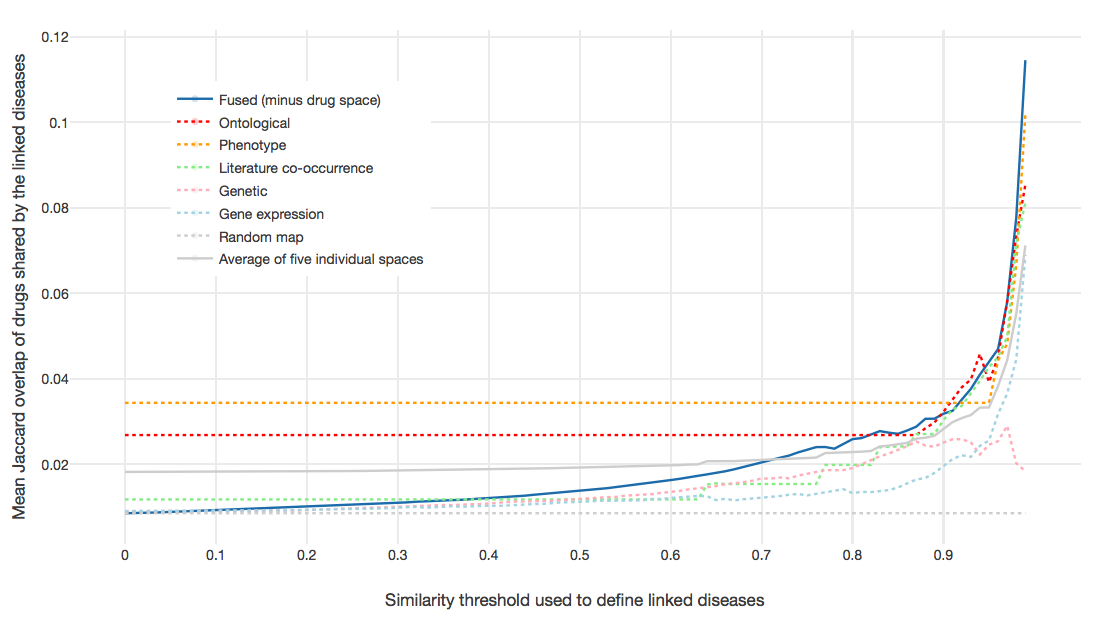
 Supplementary Fig. 7. Mean drug overlap (by Jaccard score) of diseases linked at different thresholds of similarity, approved drugs only.** The fused space has a high proportion of links which share approved drugs relative to other spaces. At the threshold of the top 6.9% most similar values (i.e. the threshold used for the disease map), the fused matrix (mean Jaccard score 0.038) is outperformed only by the ontological space (mean Jaccard score 0.040)

*
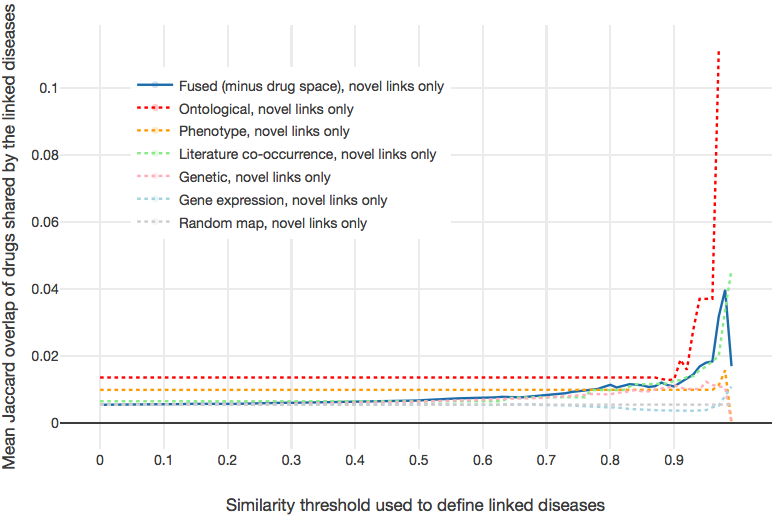
*

Approved drugs and drugs in Phase III clinical trials

*
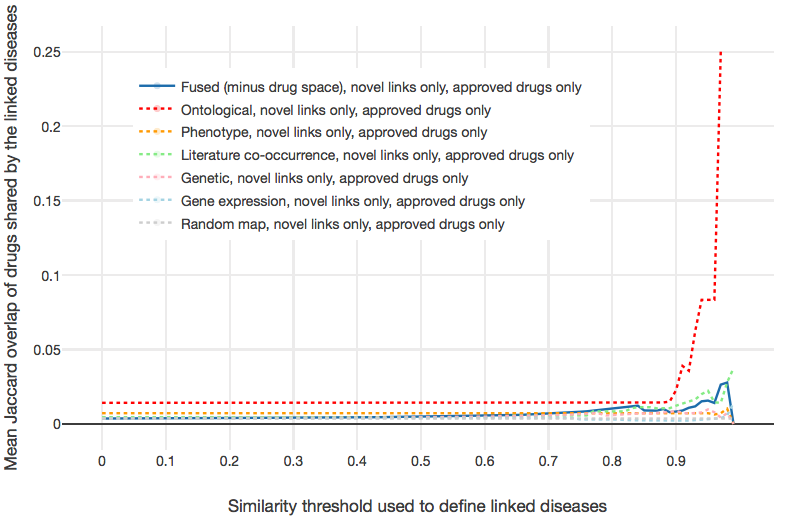
*

Approved drugs only

**Supplementary Fig. 8. Mean drug overlap (by Jaccard score) of diseases linked at different thresholds of similarity, novel links only.**

Top plot: approved drugs and drugs in phase III clinical trials

Bottom plot: approved drugs only

If only novel links (those that are in different top-level Disease Ontology classes) are considered, the ontological and literature co-occurrence spaces outperform the fused space. Examination of the novel links at a threshold of 0.9 suggests this is due to the failure of the (non-drug) fused space to identify links between neurodegenerative and mental disorders which share drugs, such as major depressive disorder/bipolar and Parkinson’s disease (identified in literature co-occurrence space) or Alzheimer’s/Parkinson’s/Huntington’s diseases and Down syndrome (identified in ontological space). Literature co-occurrence space identifies a number of additional drug-sharing pairs not in the fused space, such as polycystic ovary syndrome and type II diabetes, or cystic fibrosis and chronic obstructive pulmonary disease.

Ontological space does contain some disease pairs which are given high similarity (according to Lin’s similarity measure) despite being in different top-level classes, but as expected this number is very small compared to the other spaces, with 24 novel links at a similarity threshold of 0.9 compared to 92 for the non-drug matrix and 117 for the literature co-occurrence matrix. This decreases sharply at higher similarity thresholds, so at a similarity threshold of 0.99 there are 9 novel links in literature co-occurrence space, 2 in the non-drug matrix, and none in ontological space. This explains the high variation at the right-hand side of the plot.

**Supplementary references**

1. Schriml LM, Arze C, Nadendla S, et al. Disease Ontology: a backbone for disease semantic integration. *Nucleic Acids Res*. 2012;40(Database issue):D940-6. doi:10.1093/nar/gkr972.

2. Yu G, Wang L-G, Yan G-R, He Q-Y. DOSE: an R/Bioconductor package for disease ontology semantic and enrichment analysis. *Bioinformatics*. 2015;31(4):608-609. doi:10.1093/bioinformatics/btu684.

3. Lin D. An Information-Theoretic Definition of Similarity. *Proc Fifteenth Int Conf Mach Learn*. 1998:296-304. https://pdfs.semanticscholar.org/3216/3f8f5114beea5576c93b2ce21ec1e48988ce.pdf. Accessed May 18, 2018.

4. Hoehndorf R, Schofield PN, Gkoutos G V. Analysis of the human diseasome using phenotype similarity between common, genetic, and infectious diseases. *Sci Rep*. 2015;5(October 2014):10888. doi:10.1038/srep10888.

5. Leaman R, Lu Z. TaggerOne: joint named entity recognition and normalization with semi-Markov Models. *Bioinformatics*. 2016;32(18):2839-2846. doi:10.1093/bioinformatics/btw343.

6. Pinero J, Queralt-Rosinach N, Bravo A, et al. DisGeNET: a discovery platform for the dynamical exploration of human diseases and their genes. *Database*. 2015;2015:bav028-bav028. doi:10.1093/database/bav028.

7. Gautier L, Cope L, Bolstad BM, Irizarry RA. affy--analysis of Affymetrix GeneChip data at the probe level. *Bioinformatics*. 2004;20(3):307-315. doi:10.1093/bioinformatics/btg405.

8. NCBI. GEO2R. http://www.ncbi.nlm.nih.gov/geo/geo2r/. Accessed April 21, 2015.

9. Ritchie ME, Phipson B, Wu D, et al. limma powers differential expression analyses for RNA-sequencing and microarray studies. *Nucleic Acids Res*. January 2015. doi:10.1093/nar/gkv007.

10. Bento AP, Gaulton A, Hersey A, et al. The ChEMBL bioactivity database: an update. *Nucleic Acids Res*. 2014;42(D1):D1083-D1090. doi:10.1093/nar/gkt1031.

11. Hidalgo CA, Blumm N, Barabasi AL, Christakis NA. A dynamic network approach for the study of human phenotypes. *PLoS Comput Biol*. 2009;5. doi:10.1371/journal.pcbi.1000353.

12. Rubio-Perez C, Guney E, Aguilar D, et al. Genetic and functional characterization of disease associations explains comorbidity. *Sci Rep*. 2017;7(1):6207. doi:10.1038/s41598-017-04939-4.

13. Mehta AM, Mooij M, Branković I, Ouburg S, Morré SA, Jordanova ES. Cervical Carcinogenesis and Immune Response Gene Polymorphisms: A Review. *J Immunol Res*. 2017;2017:1-12. doi:10.1155/2017/8913860.

14. Chang M-H. Cancer prevention by vaccination against hepatitis B. *Recent Results Cancer Res*. 2009;181:85-94. http://www.ncbi.nlm.nih.gov/pubmed/19213561. Accessed November 29, 2017.
